# Supplementary material for: Multi-functional anodes boost the transient power and durability of proton exchange membrane fuel cells
Source: Nat Commun. 2020 Mar 4;11:1191. doi: 10.1038/s41467-020-14822-y (PMC7055244; doi:10.1038/s41467-020-14822-y)
Supplement: Supplementary file 1 — Supplementary Information [file 41467_2020_14822_MOESM1_ESM.pdf]

Supplementary Information

Multi-functional anodes boost the transient power and durability of proton exchange membrane fuel cells

Shen et al.

Supplementary Figures

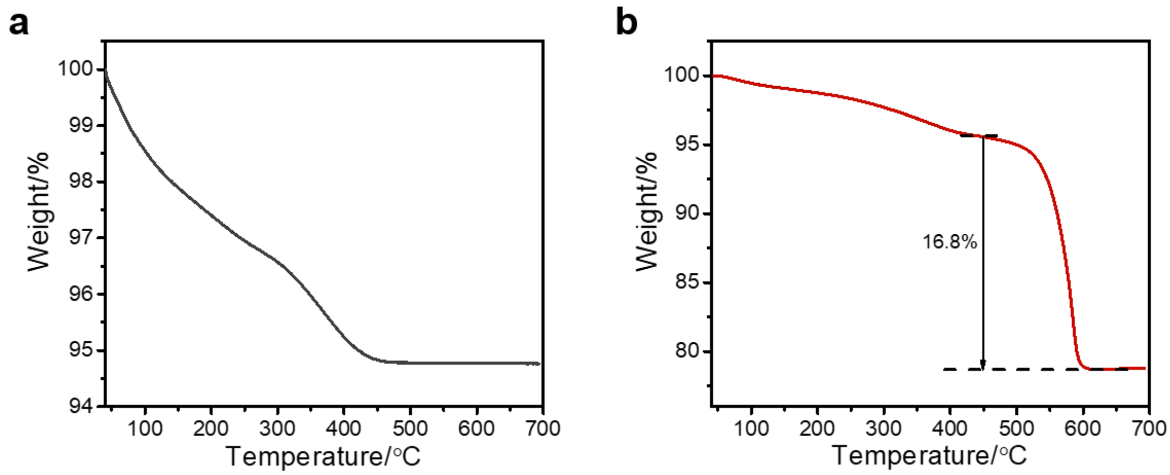

Supplementary Figure 1. TGA profiles of (a) h-WO_3 and (b) WO_3/CNTs composite in air flow.

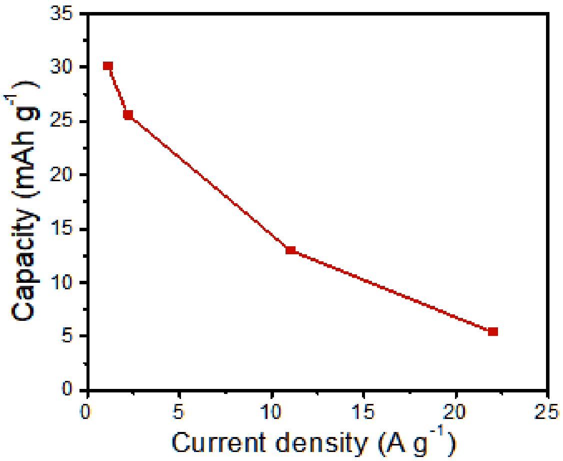

Supplementary Figure 2. Rate performance of the WO_3/CNTs electrode in 0.5 M H_2SO_4 aqueous electrolyte. Capacities of 30.1, 25.6, 13.0, and 5.4 mAh g^{-1} are delivered at current densities of 1.1, 2.2, 11, and 22 A g^{-1}, respectively.

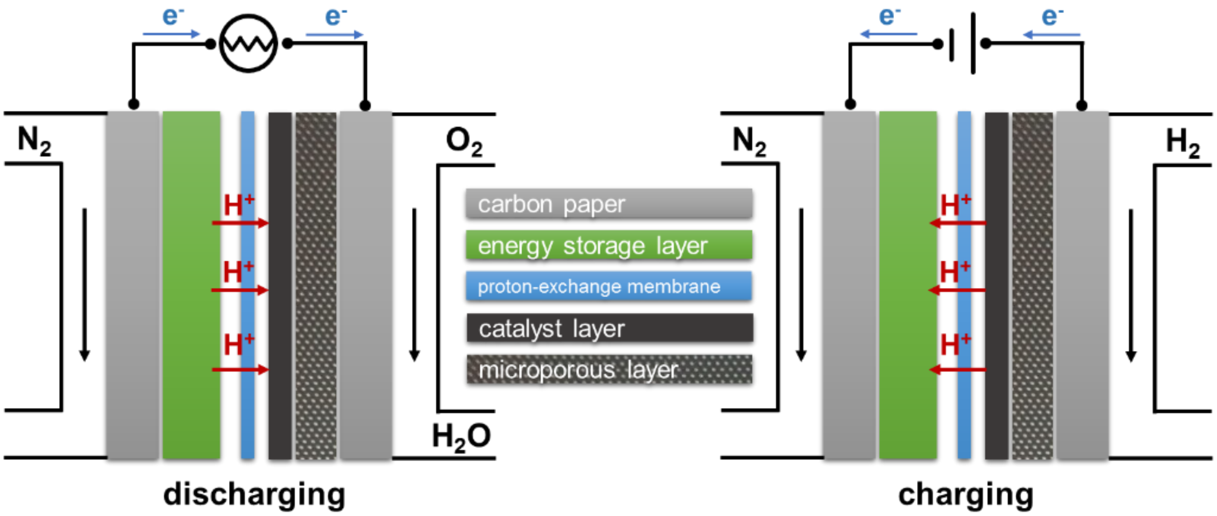

Supplementary Figure 3. Scheme of the discharging and charging process of the WO_3 electrode. During the discharge process, pre-charged H_xWO_3 anode releases protons and electrons; the protons transport through the membrane electrolyte and the electrons pass through the external circuit, both of which reach the cathode, react with O_2, and produce H_2O. To charge the WO_3 electrode, the O_2 supply in the pristine cathode is switched to H_2, and the WO_3 electrode is purged with N_2; constant current is then applied in the opposite direction to reduce WO_3 to H_xWO_3. The cells were fabricated using method similar to that of the hybrid cells, which were conducted by sandwiching a membrane (without a catalyst coating) between a WO_3 composite electrode and a prefabricated GDL, and then hot-pressed together at 130 ºC under 0.1 MPa for 1 min.

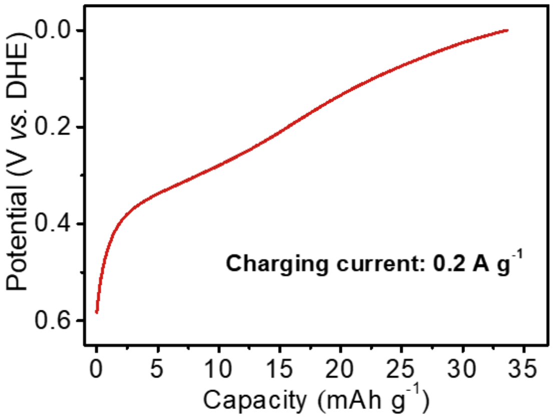

Supplementary Figure 4. Galvanostatic charge curve of the WO_3 electrode in MEA with 100 mL min^{-1} N_2 and H_2 fed to the anode and cathode, respectively.

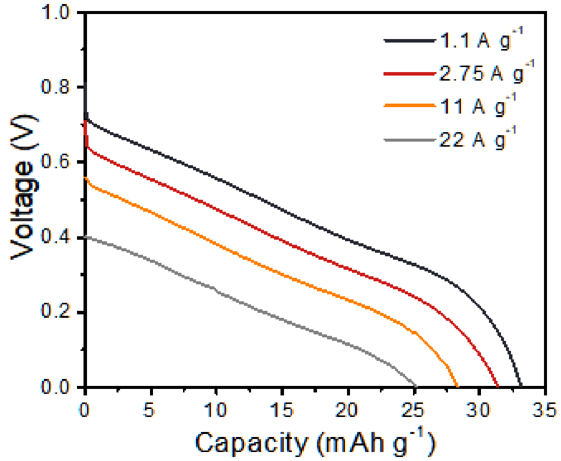

Supplementary Figure 5. Galvanostatic discharge curves of a WO_3 electrode discharged at different current densities with 100 mL min^{-1} N_2 and O_2 fed to the anode and cathode, respectively. Specific capacities of 33.2, 31.5, 28.5, and 25.6 mAh g^{-1} are obtained at a current density of 1.1, 2.75, 11, and 22 A g^{-1}, respectively.

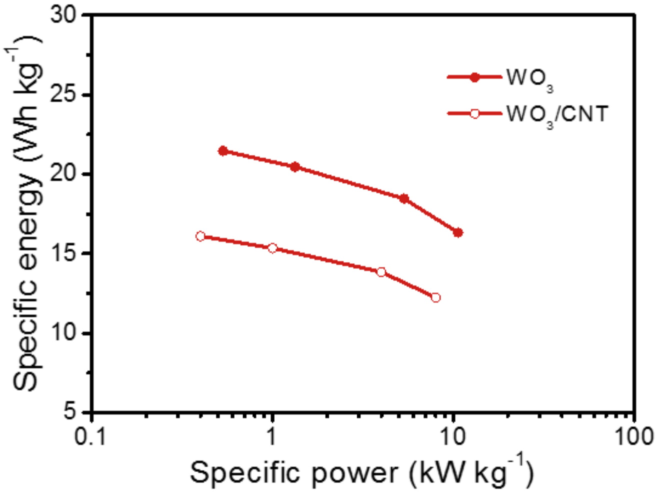

Supplementary Figure 6. Ragone plots of an air-capacitor based on the mass of WO_3/CNTs composite and WO_3, respectively. The mass of the oxygen cathode is ignored since the capacitor shares the same cathode with the fuel cell.

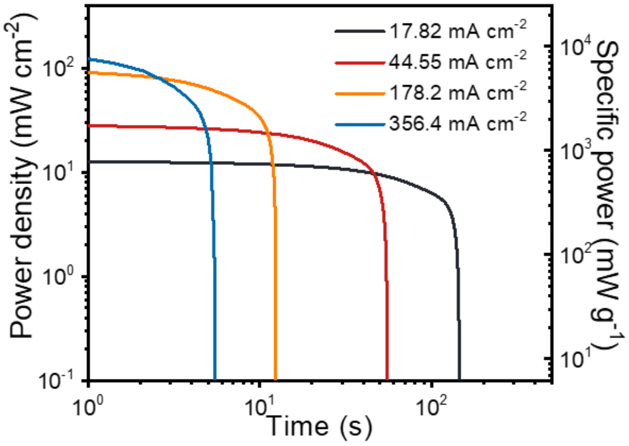

Supplementary Figure 7. Power *vs.* time plots of the electrode discharged at different current densities. The specific current densities used herein are based on the mass of WO_3.

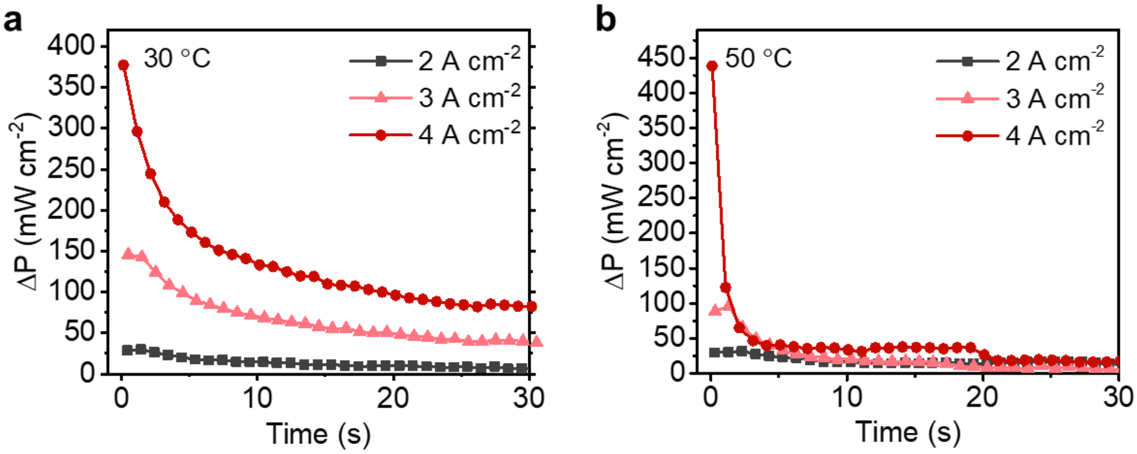

Supplementary Figure 8. ΔP of the control cell and the hybrid cell upon switching the current output from 0.05 A cm^{-2} to different current outputs at 30 °C (a) and 50 °C (b).

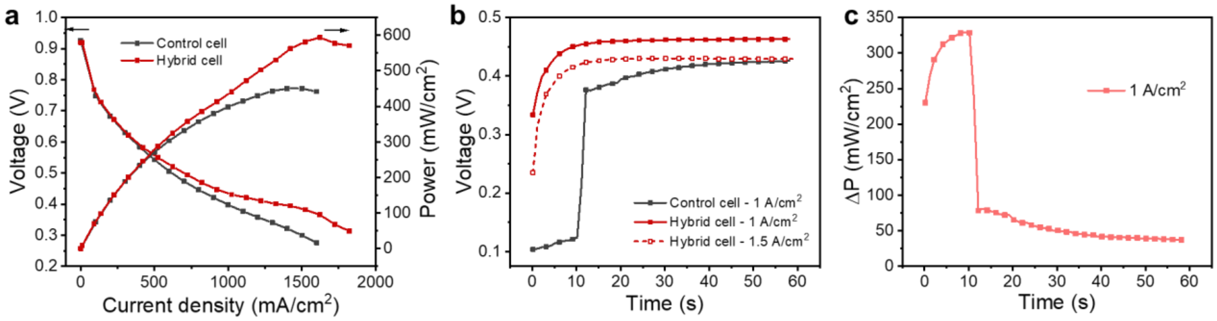

Supplementary Figure 9. (a) Polarization curves of a hybrid cell and a control cell at 70 °C, humidified H_2 (30 % relative humidity, stoichiometry = 1.5) and air (30 % relative humidity, stoichiometry = 4) were fed to the anodes and cathodes respectively. (b) Voltages of the control cell and the hybrid cell upon switching the current output from 0.05 A cm^{-2} to different current outputs at 70 °C. (c) ΔP of the control cell and the hybrid cell upon switching the current output from 0.05 A cm^{-2} to 1 A cm^{-2}.

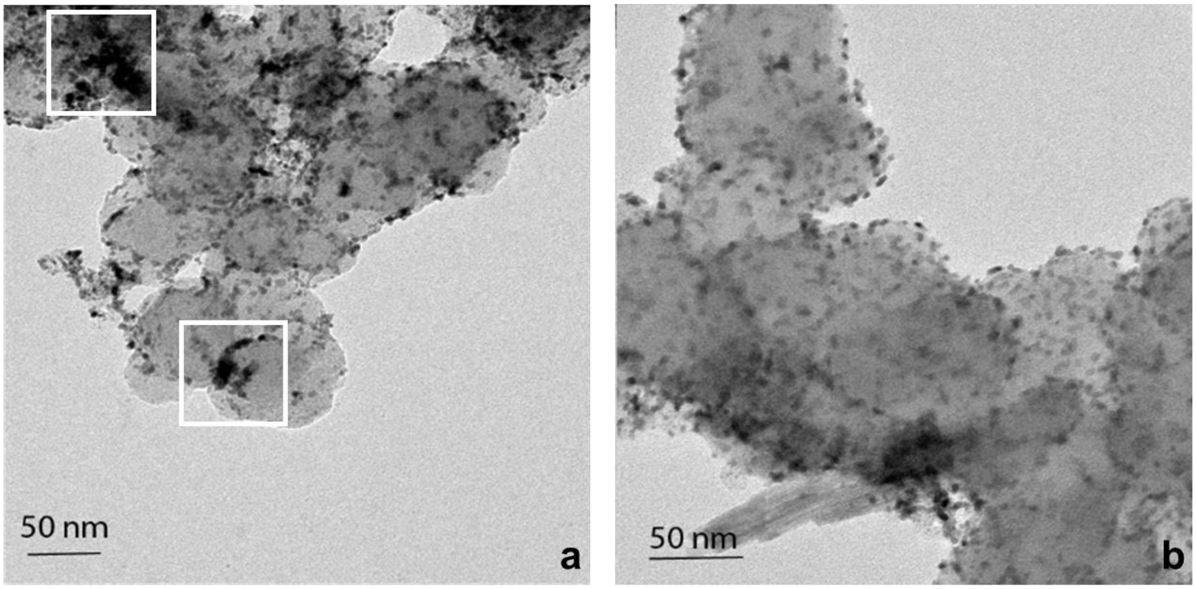

Supplementary Figure 10. Typical TEM images of the anode electrocatalysts from (a) the control cell and (b) the hybrid cell after the H_2 starvation tests. The square regions in (a) indicate aggregation of Pt nanoparticles due to anode oxidation caused by fuel starvation.

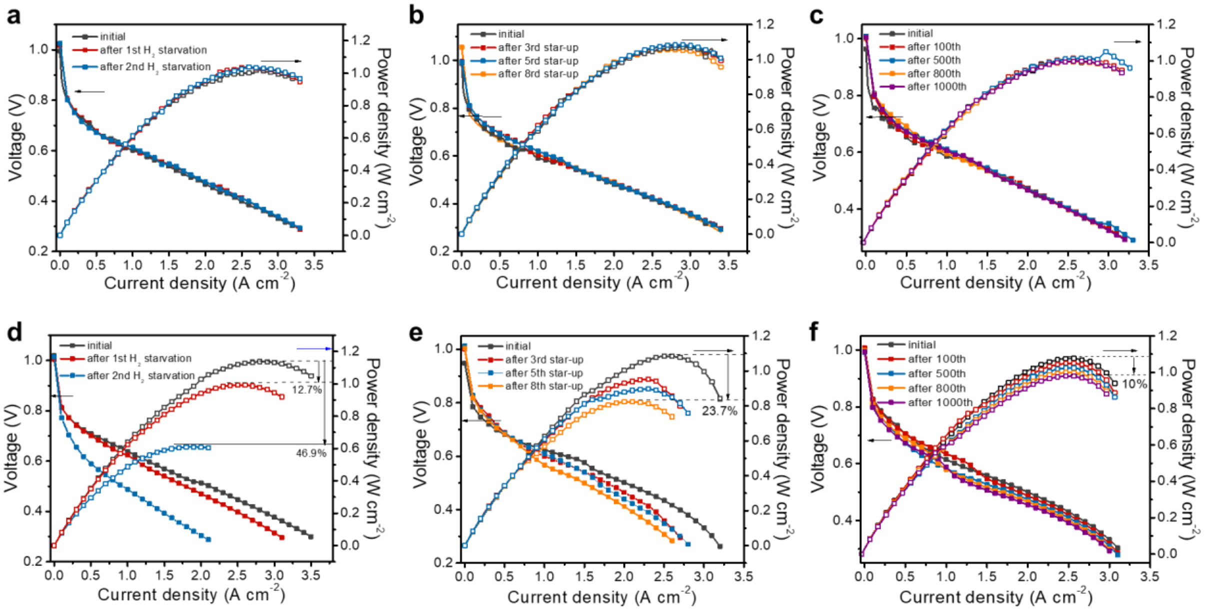

Supplementary Figure 11. Changes of polarization curves of (a-c) hybrid cell and (d-f) control cell during (a, d) hydrogen starvation, (b, e) start-up simulation, and (c, f) acceleration-deceleration cycling tests. All the steady-state performance of the hybrid cell and the control cell was tested at 50 °C, 100 % humidified H_2 (stoichiometry = 1.5) and O_2 (stoichiometry = 4) were fed to the anodes and cathodes, respectively.

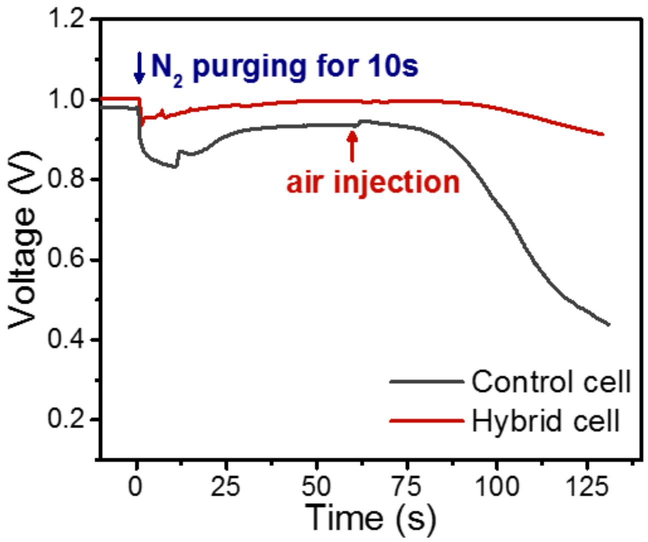

Supplementary Figure 12. OCV of the control cell and hybrid cell in response to intrusion of air prior to start-up testing. The cells were purged with N_2 for 10 s to remove most hydrogen in the anode compartments, leading to a significant OCV drop of the control cell. For the hybrid cell, the pre-charged WO_3/CNTs layer maintains the potential of anode close to 0.0 V vs. RHE, resulting in minimal change of the OCV. After air injection (1 mL at room temperature), the oxygen consumes the remaining hydrogen in the anode of the control cell and raises the anode potential, subsequently decreasing the OCV. For the hybrid cell, the anode potential experiences insignificant change, since the WO_3/CNTs layer effectively scavengers the oxygen; as a result, only a slight decrease of OCV is observed.

Supplementary Tables

Supplementary Table 1. Values of current-responsive resistor (mΩ cm)

	30 °C	50 °C
2 A cm^{-2}	8	16
	4	5
3 A cm^{-2}	14	22
	5	6
4 A cm^{-2}	26	34
	6	7

Supplementary Table 2. Fitting parameter used and parameters obtained from the fitted profiles.

Temperature (°C)	30	50
U_0 -$V_{activation}$ (V)	0.735	0.750
CRR ($\Omega \cdot cm^2$)	-5.37×10^{-5} $-7.6\times10^{-3}\times I$ $+2.8\times10^{-3}\times I^2$ $+4\times10^{-4}\times T$ (control cell)<br>$5\times10^{-4}+0.001\times I+5\times10^{-5}\times T$ (hybrid cell)	
L ($H \cdot cm^2$)	0.180 (control cell)<br>0.051 (hybrid cell)	0.050 (control cell)<br>0.014 (hybrid cell)
R_{anode} ($\Omega \cdot cm^2$)	$0.025+0.001\times I$ (control)<br>$0.023+0.001\times I$ (hybrid)	0.020 (control)<br>0.019 (hybrid)
C_{anode} (C / cm^2)	4	0.50
$R_{cathode}$ ($\Omega \cdot cm^2$)	0.089	0.087
$C_{cathode}$ (C / cm^2)	2	0.25
